# Neurofilament light chain concentration in an aging population

**DOI:** 10.1007/s40520-021-02054-z

**Published:** 2022-01-11

**Authors:** Aurélie Ladang, Stéphanie Kovacs, Laetitia Lengelé, Médéa Locquet, Jean-Yves Reginster, Olivier Bruyère, Etienne Cavalier

**Affiliations:** 1 Clinical Chemistry Department, CHU de Liège, University of Liège, Avenue de L’Hopital, 1, 4000 Liège, Belgium; 2 WHO Collaborating Centre for Public Health Aspects of Musculoskeletal Health and Aging, Division of Public Health, Epidemiology and Health Economics, University of Liège, Liège, Belgium; 3 Physical, Rehabilitation Medicine and Sports Traumatology, SportS2, CHU de Liège, University of Liège, Liège, Belgium

**Keywords:** Neurology, Blood-based biomarkers, NF-L, Reference ranges, Aging

## Abstract

**Background:**

Neurofilament light chain (NF-L) concentration is recognized to be modified in neurological diseases and traumatic brain injuries, but studies in the normal aging population are lacking. It is, therefore, urgent to identify influencing factors of NF-L concentration in the aging population.

**Method:**

We assessed NF-L concentration in sera of a large cohort of 409 community-dwelling adults aged over 65 years. We studied the association between NF-L and various physiological factors but also with self-reported comorbidities or life-style habits.

**Results:**

We showed that NF-L concentration in serum was tightly associated with cystatin C concentration (*r* = 0.501, *p* < 0.0001) and consequently, to the estimated glomerular filtration rate (eGFR) (*r* = − 0.492; *p* < 0.0001). Additionally, NF-L concentration was dependent on age and body mass index (BMI) but not sex. Among the self-reported comorbidities, subjects who reported neurological disorders, cardiovascular diseases or history of fracture had higher NF-L concentration in univariate analysis, whereas it was only the case for subjects who reported neurological disorders in the multivariate analysis. NF-L concentration was also increased when Mini-Mental State Examination (MMSE) was decreased (≤ 25 points) but not when geriatric depression score (GDS) was increased (> 5 points) in both univariate and multivariate analysis. Finally, we are providing reference ranges by age categories for subjects with or without altered renal function.

**Conclusion:**

NF-L concentration in the aging population is not driven by the increasing number of comorbidities or depression. Yet, NF-L blood concentration is dependent on kidney function and NF-L interpretation in patients suffering from renal failure should be taken with caution.

## Introduction

The diagnostic and follow-up of neurological disorders is based mainly on clinical features and imaging techniques, as there are only a few blood-based biomarkers available (such as neuron-specific enolase or S100B). Indeed, brain- and neuron-specific proteins usually have a low blood concentration as the blood–brain barrier (BBB) creates a separation in between compartments [1]. With the emergence of new technologies such as the single-molecule array (SiMoA°), new neurology biomarkers are emerging [2]. These technologies with improved sensitivity are able to measure proteins at subfemtomolar concentrations [3]. Therefore, many proteins, previously only studied in the cerebrospinal fluid (CSF), are now measurable in blood, resulting a less invasive and traumatic testing procedure for the patient.

Among these neurological blood-based biomarkers, neurofilament light chains (NF-L) are very promising. Neurofilaments are cytoskeletal proteins only expressed in neurons. They are part of the axons and participate in establishing axonal diameter. Neurofilaments are composed of light, medium and heavy chains as well as a fourth subunit that varies depending on the neuron location. Neurofilaments are released in the CSF in cases of central or peripheral neuronal damage [4].

Due to the high specificity of NF-L to the nervous system, many studies are now trying to establish the NF-L context for use in daily clinical practice. First proposed as a marker for Alzheimer’s disease or amyotrophic lateral sclerosis [5], it is now recognized that NF-L concentration is altered in many pathologies affecting the nervous system [4]. Indeed, its concentration has been reported as modified in chronic neurodegenerative disorders (multiple sclerosis, Alzheimer’s disease, Parkinson disease,…) [6–8], in acute neuronal damage linked to hypoperfusion of the brain (ischemic stroke or cardiac arrest) [9, 10] and in traumatic brain injuries such as sport concussions [11]. Therefore, NF-L is suggested to be a pan-biomarker of chronic or acute neurological damage. The two major applications that are now emerging are, first, in the follow-up of long-term diseases such as multiple sclerosis [12] and, second, in the differentiation between neurological and psychological disorders [13].

As NF-L is almost launched as a biomarker in daily practice, many questions are arising regarding its expression in the general population. Studies reporting NF-L concentration in the normal population are rare [14, 15] and many studies highlight the lack of data on the normal aging population as well as the urgent need to assess NF-L’s analytical matters [16]. Therefore, we decided to study NF-L concentration in a large cohort of community-dwelling aging individuals to determine influencing factors and reference ranges for NF-L concentration in serum.

## Materiels and methods

### Sample collection

All subjects participated in a long-term prospective study called the SarcoPhAge study (for sarcopenia and physical impairment with advancing age). This study followed more than 500 individuals aged 65–92 years over 5 years to evaluate the quality of life and the consequences of sarcopenia in older community-dwelling Belgian subjects. The complete methodology has been described elsewhere [17]. Briefly, cognitive function was evaluated by answering the 30 questions of the Mini-Mental State Examination (MMSE), and the depression was assessed by the geriatric depression score (GDS). The comorbidities considered were as follows: asthma, ear–-nose–throat (ENT) inflammation, cardiac troubles, hypertension, history of ischemic stroke, gastrointestinal troubles, diabetes, back pain, neurological troubles, migraines, allergy, cutaneous diseases, history of cancer, prostate troubles, osteoporosis, arthritis and history of fracture. The only exclusion criteria for the SarcoPhAge study were limb amputation and body mass index > 50 kg/m^2^. We analysed all the available sera collected at the time of inclusion (*n* = 409). The SarcoPhAge study was approved by the Ethical Committee of the CHU de Liège (2012/277).

### Laboratory analysis

Serum NF-L was analysed with SiMoA° technology on an SR-X platform (Quanterix, USA) following the manufacturer’s instructions. The lower limit of quantification of 0.316 pg/mL was defined by Quanterix° as the lowest concentration with a coefficient of variation (CV) below 20%. The CV for an internal control at 5.08 pg/mL was estimated at 10.7% by Quanterix° and at 16.1% by our laboratory. Serum standardized cystatin C was measured with an immunoturbidimetric assay (Tina-quant Cystatin C Gen.2 assay) on Cobas C6000 (Roche, Germany). The estimated glomerular filtration rate was calculated using the CKD-EPI equation for cystatin C only [18].

### Statistical analysis

First, the distribution of all the continuous variables was checked using the Shapiro–Wilk test. For descriptive statistics, the results of continuous variables were expressed as the mean with standard deviation (SD) as well as median with interquartile range (IQR). For qualitative data, the results were expressed as absolute (*n*) and frequencies (%). Linear regression and Spearman correlation tests were used to investigate the association between two continuous variables. Differences in NF-L concentration according to categorical data were assessed using the Kruskall–Wallis test. For the multivariate model, multiple regression with NF-L concentration as dependent variable was performed according to Altman et al. [19]. All the independent variables were entered into the model in one single step without checking for level of significance. Coefficient of determination R2 was adjusted for the number of independent variables in the regression model. The level of statistical significance was set at 5%. All statistical analyses were performed with Medcalc° (Medcalc software, Belgium).

In figures displaying the qualitative data, medians as well as 25–75 percentiles are represented by horizontal lines, whereas each single result is represented by a dot. For continuous data, the black line represents the regression line.

Reference ranges were calculated based on the normal distribution method on back-transformed data after NF-L logarithmic transformation. At first, all subjects except subjects who reported neurological disorders or who had an MMSE score ≤ 25 were included. Then, outliers were excluded once according to Tukey’s method.

## Results

### Influencing factors of NF-L concentration: univariate analysis

**Fig. 1:**
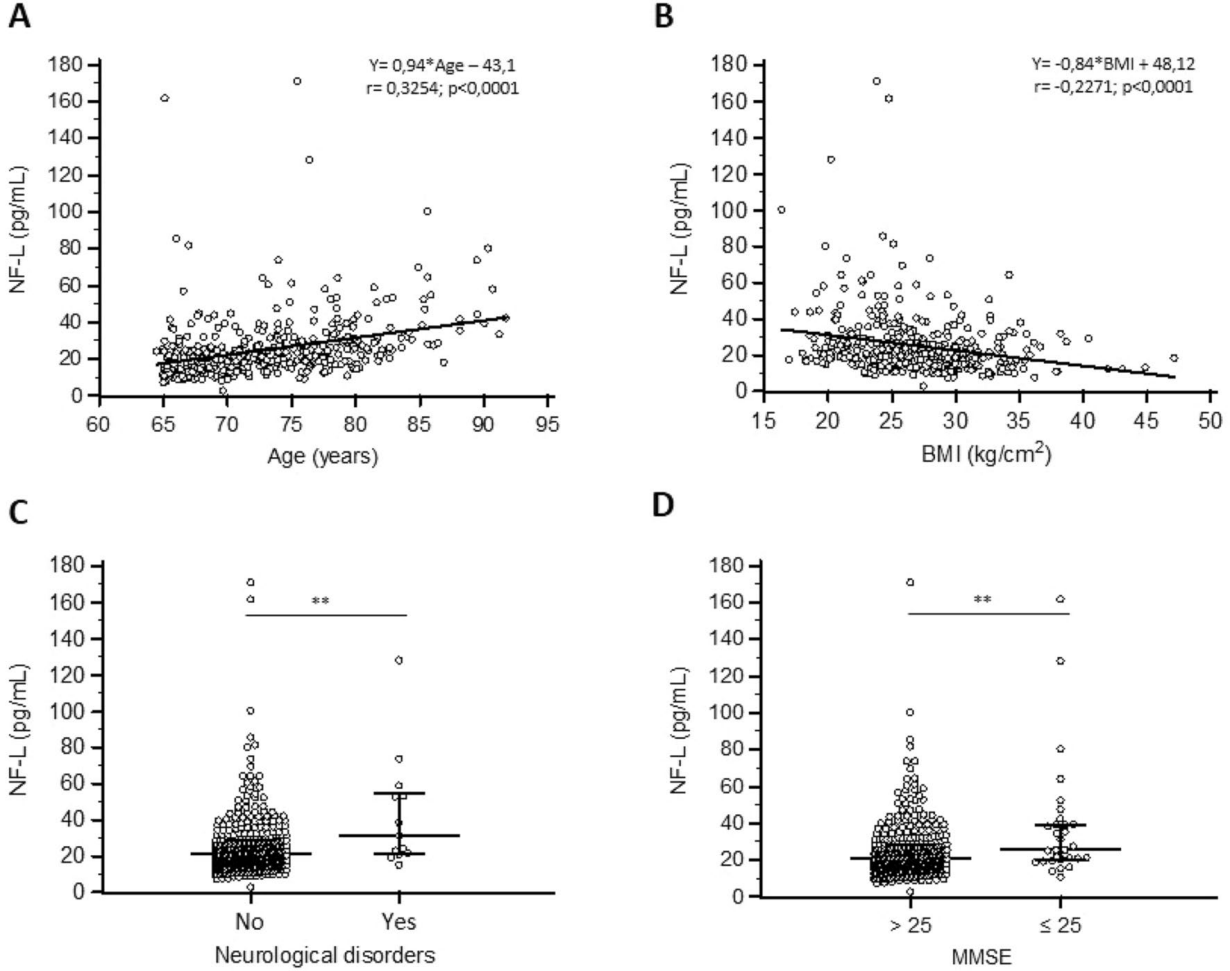
NF-L and influencing factors. (**A**) and (**B**): correlation and regression of NF-L with age or BMI. Continuous line represents regression equation as mentioned in the graph. *r* represents correlation coefficient. (**C**) and (**D**): Scatterplot of NF-L of subjects reporting or not neurological disorders (*p* = 0.007) and subjects with MMSE above or below 25 (*p* = 0.001)

The influencing factors evaluated in this study are shown in Table 1. We evaluated the following three demographic parameters: age, sex and body mass index (BMI). A positive correlation was found between age and NF-L concentration (Spearman’s rho: 0.325; *p* value: < 0.0001) and a negative one between BMI and NF-L (Spearman’s rho: − 0.227; *p* value: < 0.0001) (Fig. 1). When age and BMI were categorized according to the subgroups mentioned in Table 1, a significant difference was observed between groups (*p* value: < 0.0001; 0.0008, respectively). The NF-L concentration did not differ between the sexes (Table 1).

**Table 1:** Univariate analysis of NF-L concentration with influencing factors

Variables	*n* (%)	Median NF-L (IQR)	Mean NF-L (SD)	*p* value
Total	409 (100)	21.3 (13.6)	25.5 (17.4)	n.a
Demographic				
Age				< 0.0001***
< 70 years	155 (37.9)	17.1 (11.4)	20.7 (16.1)	
70–74 years	107 (26.2)	21.0 (10.1)	23.8 (11.1)	
75–79 years	91 (22.2)	24.8 (12.9)	29.0 (21.6)	
≥ 80 years	56 (13.7)	30.9 (20.0)	36.6 (17.5)	
Sex				0.586
M	173 (42.3)	20.8 (12.3)	24.6 (14.3)	
F	236 (57.7)	21.6 (14.5)	26.1 (19.4)	
BMI				0.0008***
< 20	23 (5.6)	21.2 (26.7)	32.4 (22.6)	
20–24	134 (32.8)	23 (16.5)	29.8 (23.5)	
25–29	163 (39.9)	21.7 (12.2)	23.5 (12.3)	
≥ 30	89 (21.8)	18.6 (13.3)	20.9 (10.0)	
Measured comorbidities				
Renal function				< 0.0001***
GFR (CKD-EPI Cys) ≥ 60	254 (62.1)	18.5 (9.5)	20.3 (9.7)	
GFR (CKD- EPI Cys) < 60	155 (37.9)	28.4 (17.7)	34.0 (23.1)	
Self-reported comorbidities				
Number of comorbidities				0.574
0–2	102	21.2 (14.4)	25.7 (14.2)	
3–5	202	21.4 (12.2)	24.5 (14.9)	
> 5	105	21.1 (15.9)	27.2 (23.7)	
Diabetes				0.95
No	342 (83.6)	21.2 (12.8)	25.3 (16.3)	
Yes	67 (16.4)	21.8 (17.2)	26.8 (22.6)	
Cardiovascular disease				0.001**
No	312 (76.3)	20.8 (12.2)	23.8 (13.8)	
Yes	97 (23.7)	24.6 (18.3)	31.1 (25.2)	
Hypertension				0.194
No	235 (57,5)	21.6 (14.0)	26.6 (18.5)	
Yes	174 (42.5)	20.7 (12.2)	24 (15.7)	
History of fracture				0.029*
No	183 (44.7)	20.2 (12.1)	23.7 (13.4)	
Yes	226 (55.3)	23.4 (13.5)	27 (20.0)	
Self-reported lifestyle habits				
Smoking				
No	373 (91.2)	21.3 (13.2)	25.1 (16.1)	0.643
Yes	36 (8.8)	21.3 (16.3)	29.8 (27.8)	
Alcohol				0.743
No	204 (49.9)	21.2 (14.3)	25.0 (16.2)	
Yes	205 (50.1)	21.4 (13.5)	26 (18.6)	
Number of drugs				0.1316
0–3	108 (26.4)	19.4 (12.5)	24.0 (15.6)	
4–6	154 (37.7)	21.6 (13.0)	25.2 (15.2)	
≥ 7	147 (35.9)	22.8 (14.8)	27.0 (20.6)	
Neurological impairments				
Self-reported neurological troubles				0.007**
No	396 (96,8)	21.1 (13.2)	24.9 (16.5)	
Yes	13 (3.2)	31.5 (33.1)	43.1 (31.4)	
MMSE				0.001**
≤ 25	31 (7.6)	25.9 (18.6)	38.1 (32.6)	
> 25	378 (92.4)	21 (12.9)	24.5 (15.2)	
GDS				0.295
< 5	274 (67.0)	21 (12.5)	23.9 (13.0)	
5–9	98 (24.0)	22.4 (16.3)	28.1 (21.2)	
> 9	37 (9.0)	23 (10.0)	30.2 (29.7)	

Stars are showing statistically significant results (*p* < 0.05)

Because extended comorbidities have been suggested to have an impact on NF-L concentrations, we assessed NF-L levels according to self-reported comorbidities at time of inclusion in the study. Only those subjects who reported neurological troubles, cardiovascular diseases or history of fracture were found to have a significantly different NF-L concentration compared to subjects who did not report the comorbidity (*p* value: 0.007; 0.001 and 0.029, respectively) (Table 1). We also investigated self-reported lifestyle habits such as smoking or alcohol consumption. Subjects who reported drug or alcohol consumption had no difference in NF-L concentration compared to subjects who did not report these lifestyle habits (Table 1). Finally, NF-L concentration was independent of the number of self-reported comorbidities or the number of medical drugs taken (Table 1).

**Fig. 2:**
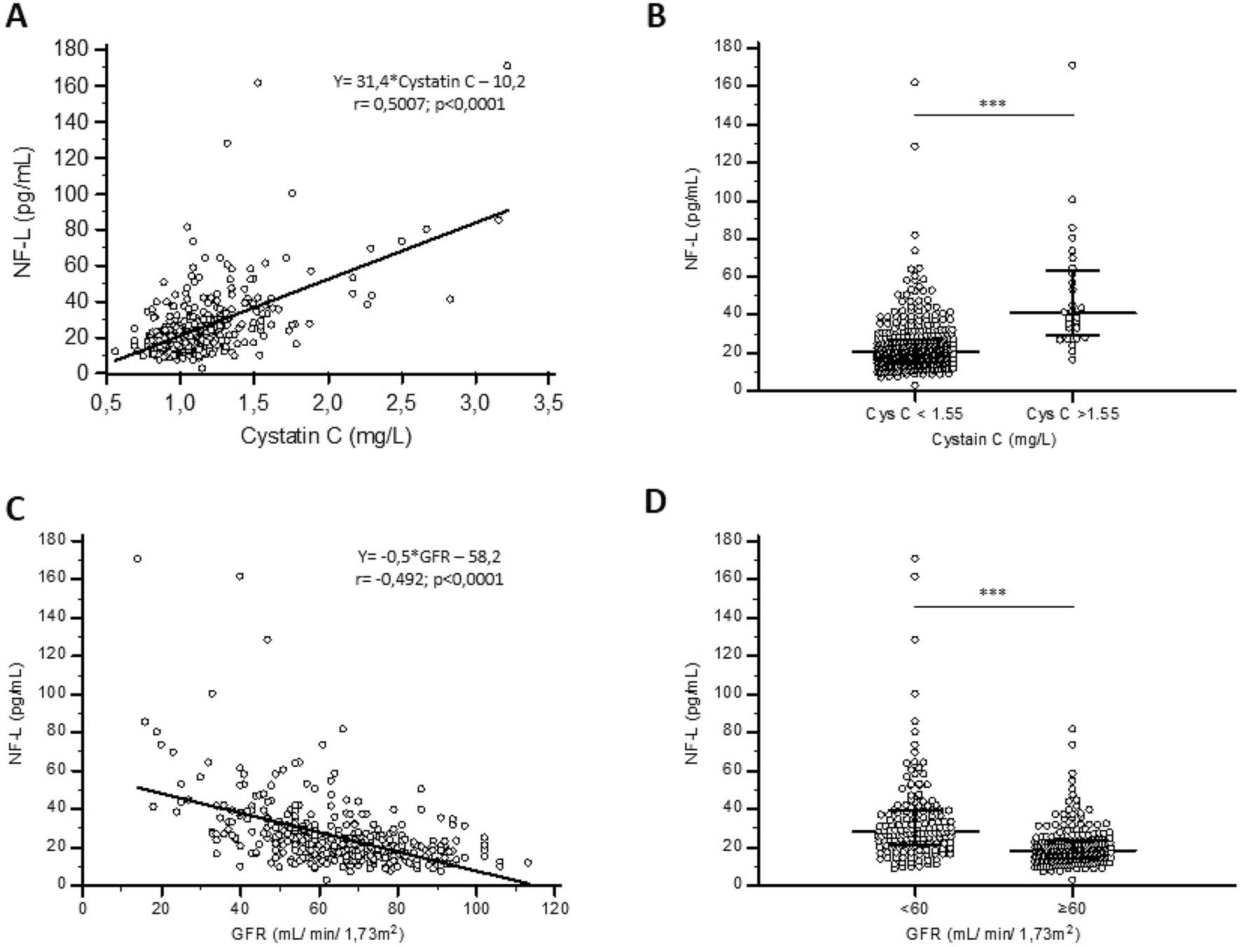
NF-L concentration in renal function. (**A**) and (**C**) Correlation and regression of NF-L with Cystatin C or GFR. Continuous line represents regression equation as mentioned in the graph. *r* represents correlation coefficient. (**B**) and (**D**) Scatterplot of NF-L of subjects with or without altered renal function based on Cystatin C (*p* < 0.0001) or GFR levels (*p* < 0.0001)

However, it is important to mention that the NF-L concentration was highly correlated with kidney function as assessed by cystatin C (Spearman’s rho: 0.501; *p* value: < 0.0001). We further calculated eGFR based on the CKD-EPI equation for cystatin C. This equation also takes into account age and sex as factors [18]. A very good correlation was also observed between NF-L concentration and eGFR (Spearman’s rho: − 0.492; *p* value: < 0.0001) (Fig. 2). When patients were separated into normal eGFR (≥ 60 mL/min/1.73m^2^) or decreased kidney function (eGFR < 60 mL/min/1.73m^2^), we found that the median NF-L concentration was 1.5-fold higher in subjects with altered kidney function (18.5 ng/mL and 28.4 ng/mL, respectively; *p* value: < 0.0001) (Table 1 and Fig. 2).

We also checked all the available neurological data. Subjects who self-reported neurological troubles or with MMSE score below or equal to 25 had significantly higher NF-L concentration than their counterparts (*p* value: < 0.007 and 0.001, respectively) (Table 1 and Fig. 1). Importantly, subjects with GDS > 5 points did not show higher NF-L concentration compared to the others (*p* value: 0.295) (Table 1).

### Influencing factors of NF-L concentration: multivariate analysis

To define the most relevant comorbidities, we performed a multiple regression analysis with all the comorbidities for which NF-L was significantly differentially expressed in subjects who reported the comorbidity compared to the others as follows: cystatin C, BMI, age, self-reported neurological troubles, MMSE, history of fracture and cardiovascular disease. eGFR was not included in the model because the eGFR equation we used is based on other variables of the model (cystatin C and age). In this model, NF-L was independently associated with cystatin C, BMI, age, self-reported neurological troubles and MMSE but not with history of fracture or cardiovascular disease (Table 2).

**Table 2:** Multivariate analysis of NF-L concentration adjusted for influencing factors that were found significantly different in the univariate analysis

Variables	*r*_partial_	*p* value
Cystatin C	0.5343	< 0.0001***
BMI	− 0.2805	< 0.0001***
Age	0.1601	0.0013**
Neurological troubles	0.1527	0.0021**
MMSE	0.1096	0.028*
History of fracture	0.094	0.0597
Cardiovascular disease	0.0298	0.5511

Stars are showing statistically significant results in the multivariate analysis (*p* < 0.05)

### Reference ranges in an aging population

To establish reference ranges in aging individuals, we excluded individuals who reported neurological troubles or showed a MMSE score ≤ 25. The reference ranges are shown in Table 3 and were established according to age and according to KDIGO cut-offs defined for G3a and G3B altered kidney function [20]. Noticeably, the upper limit for individuals older than 75 years is 40.1 ng/mL compared to 36.2 ng/mL for individuals between 65 and 75 years old. When looking at renal function, the upper limits were 37.7 ng/mL; 57.3 ng/mL; 97.8 ng/mL for subjects with eGFR above 60; eGFR between 45 and 60 or eGFR below 45, respectively. These data highlight that renal function had a much greater impact on NF-L concentration than age.

**Table 3:** NF-L reference ranges according age and renal function

Age	*n*	Mean	Lower limit (95% CI)	Upper limit (95% CI)
GFR ≥ 60 mL/min/1.73 m^2^				
≥ 65 years	233	18.2	8.8 (8.2–9.4)	37.7 (35.2–40.4)
≤ 75 years	177	17.4	8.4 (7.7–36.2)	36.2 (33.4–39.2)
> 75 years	56	20.8	10.8 (9.5–12.3)	40.1 (35.3–45.6)
45 ≤ GFR < 60 mL/min/1.73 m^2^				
≥ 65 years	95	25.0	10.9 (9.6–12.3)	57.3 (50.6–64.9)
GFR < 45 mL/min/1.73 m^2^				
≥ 65 years	36	37.3	12.9 (10.1–16.5)	97.8 (76.4–125.3)

## Discussion

In this study, we report NF-L concentrations in a large and well-described cohort of Belgian community-dwelling individuals. We confirmed several already known influencing factors such as age or BMI. Indeed, NF-L has repeatedly been reported to be increased by aging [1, 14, 15]. This phenomenon has been partially associated with normal brain atrophy occurring with aging [14]. Additionally, different reports have mentioned the possibility that NF-L concentration could face a larger distribution in the aging population which was not the case in our study [1, 14]. However, because our study does not include young individuals, our observations should be bias by the narrow age range. An increased number of comorbidities has also been proposed to be the reason for the larger NF-L distribution in aging population [1, 14]. In our study, the number of comorbidities did not impact NF-L concentration but kidney function did. As kidney function decreases with age, it was important to show in a multivariate analysis that NF-L was independently associated with both age and kidney function. Thus, not only age but also age-related decreased renal function should be considered as the cause for the larger NF-L distribution previously reported in aging population.

However, we could not find correlations with some comorbidities previously reported. Indeed, a history of ischemic stroke did not result in an increased NF-L concentration although this has been previously reported [21]. Apart from a misreporting of the comorbidity, this might be due to a timing issue. Indeed, timing between ischemic stroke and inclusion in our study was not considered. This probably means that NF-L returns to its normal level after a certain period of time. Additionally, we could not find any association between NF-L concentration and diabetes or hypertension. Yet, with hypertension, we meant “people currently treated and followed-up for hypertension”. Thus, these subjects might have had a normal blood pressure under medication at the time of inclusion. The same comment is also true for diabetes patients. No data regarding the type of diabetes or HbA1C were considered. Yet, our data show that when correctly followed-up, neither a single comorbidity (apart from kidney function) nor the increasing number of comorbidities influenced NF-L concentration.

Regarding the association between kidney function and NF-L concentration, a correlation was established in a cohort of healthy and diabetic subjects [22]. Renal function was also shown to be an independent predictor in a multivariate model of NF-L concentration of another diabetes cohort [23] and NF-L has been reported to be increased in end-stage renal disease [24]. The fact that kidney function was so poorly reported as an influencing factor is probably due to two circumstances. First, it was not assessed in most cohorts of normal individuals [14, 15]. Second, kidney failure might be an exclusion criterion for some studies [25].

In this study, the association between renal function and NF-L is based on cyctatin C. Cystatin C was initially chosen because creatinine is known to be influenced by muscle mass and this cohort was initially designed to evaluate prevalence of sarcopenia. Cystatin C is as good as or even better than creatinine in estimating GFR [26] although cystatin C is less commonly used on a routine basis because it is more expensive for patients. In previous studies [22, 24], renal function was assessed based on creatinine rather than cystatin C emphasizing that our conclusions are driven by renal function rather than by the method that estimates renal function.

How kidney function alters NF-L concentration is still unclear and NF-L clearance has not been evaluated so far. The first hypothesis could be that elevated NF-L concentration is due to decreased clearance of NF-L by the kidney. Yet, given that NF-L is a 62 kDa protein, it should not be filtered at the glomerular level. Moreover, given the high specificity of neurofilaments to the nervous system, the release of NF-L in cases of kidney trauma is unlikely. Therefore, a third explanation could be that the NF-L antibody not only recognizes the intact molecule but also smaller NF-L fragments. These fragments could arise from neuronal damage itself or from the metabolism of NF-L. In that case, these fragments would be filtered at the glomerular level and would accumulate in case of altered glomerular filtration. This is a known phenomenon for many proteins such as parathyroid hormone. However, investigations must be made to confirm this hypothesis.

Whether kidney function also alters CSF NF-L concentration is unknown. As the NF-L half-life in blood and CSF is also unknown [27], we do not know if Nf-L crosses the BBB as an intact form or in smaller fragments. Furthermore, whether crossing the BBB is dependent on the integrity of this barrier is also still unclear [1]. Indeed, in multiple sclerosis where chronic inflammation leads to a disruption of the BBB, NF-L does not systematically correlate with BBB integrity [28, 29]. However, CSF and blood NF-L concentrations are usually well correlated but might signal different clinical outcomes [30].

No reference values were previously reported in cases of altered renal function. However, two studies have already reported age-related reference ranges. The first one by Khalil et al. is based on a cohort of 335 Caucasian subjects aged 45–85 years in which exclusion criteria were history of stroke or dementia [14]. The second one is based on a smaller cohort of 165 Chinese individuals aged between 20 and 80 years with no history of neuropsychiatric disease, stroke or dementia [15]. Both studies found a need for partitioning according to age but none assessed kidney function. Consequently, our upper reference limits are lower than those mentioned for the same age range in these earlier studies, none of which excluded subjects with altered renal function. Noticeably, the selection of healthy geriatric subjects is a matter of debate [31]. Indeed, it has been shown that, given the prevalence of comorbidities in the 70–80 year-old population, 9 out of 10 people should be excluded from reference ranges [32]. In this cohort, subjects with 2 or fewer comorbidities represents a quarter of the cohort (102 individuals). Therefore, the number of “healthy subjects” is even lower and certainly too low to fit the 120 individuals recommended by the CLSI guidelines. Consequently, given that our exclusion criteria are the known influencing factors of NF-L concentrations, we chose the term “normal aging” population rather than “healthy” population.

Our study still faces some limitations. First, this study focuses on the aging population. Therefore, data need to be confirmed in the younger population as well. Second, self-reporting of comorbidities is not the strongest way to define clinical features and the prevalence of some comorbidities or lifestyle habits may have been over- or underreported. Third, this study does not provide any information on analytical and preanalytical issues linked to NF-L measurements. Finally, cognitive function was only assessed through self-reporting of neurological troubles and MMSE score. Yet, as recruitment was made through advertisement in the local press, the enrolled population was still able to read the press and thus should be only weakly cognitively impaired. Still, even when neurological troubles are only self-reported, we observed that NF-L might help in distinguishing neurological troubles from depression (in this case, based on GDS). Even if these data are not strong enough to conclude a clinical indication for NF-L testing, our observations are perfectly in line with the literature [33, 34]. Indeed, the distinction between neurological and psychological troubles is currently one of the main indications for NF-L measurement [13].

In conclusion, our data show that the NF-L specificity for nervous system is challenged by its underestimated association with kidney function, thus, limiting its interpretation in subjects with kidney failure.
